# Role of Sensitization and Awareness Program on Knowledge, Attitude, Views, and Practice of Self-Medication Among MBBS Students in a Medical College of Bihar

**DOI:** 10.7759/cureus.40774

**Published:** 2023-06-22

**Authors:** Manish Kumar, Sukalyan S Roy, Saajid Hameed, Adil A Shakur, Lalit Mohan, Harihar Dikshit

**Affiliations:** 1 Department of Pharmacology, Indira Gandhi Institute of Medical Sciences, Patna, IND

**Keywords:** views, self-medication, practice, mbbs students, knowledge, attitude

## Abstract

Aims

Self-medication is an essential component of self-care; however, its use has significantly increased. Its practice has many risks such as wrong diagnosis, adverse drug reactions, antimicrobial resistance, etc. Being future doctors, self-medication has a special impact on MBBS students. Henceforth, the present study was undertaken to sensitize MBBS students in a medical college in Bihar and to analyze its role in different aspects of self-medication.

Methods and material

This was a cross-sectional, questionnaire-based study. The questionnaire was circulated to MBBS students of all the phases. After collecting the responses, scoring and grading was done and then a sensitization and awareness program was conducted through different modes and medium. After three months the same questionnaire was distributed, and their response was again collected.

Statistical analysis used

With an expected 40% prevalence, the minimum sample size needed to attain a power of 95% and an alpha value of 0.05 was calculated to be 201. Statistical Package for Social Sciences version 16 was used for data analysis. The chi-square test was used to see the association in responses obtained, before and after sensitization. A P-value with ≤0.05 was considered statistically significant.

Results

The questionnaires were circulated among 439 students. On comparing the grades, we found that phase III students comparatively had better knowledge regarding different aspects of self-medication. On analyzing different aspects of self-medication, we found that there was a significant improvement in many aspects post-sensitization.

Conclusions

MBBS students are future doctors, hence they should be more educated about the pros and cons of self-medication. This study showed that the perception of participants improved after our educational activities. We hope that after becoming aware, these young budding doctors will spread awareness, which, in turn, will benefit society. Additionally, we hope that this study will have an impact on students from different medical colleges and even healthcare providers, promoting responsible self-medication practices when necessary.

## Introduction

Self-medication is defined as the “selection and use of medicines/medicinal products including herbal and traditional products by individuals to treat self-recognized illness or symptoms, or the intermittent or continued use of medication(s) prescribed by the physician for chronic or recurring diseases or symptoms” [1]. It is an essential and pivotal component of self-care; however, with the development and discovery of new drugs, the use of self-care in the form of self-medication has increased which could be attributed to several reasons such as increased availability of drug information on the internet search platforms and social media, suggestions from an advertisement in newspapers, advice given by families, friends, neighbors and the pharmacist, sharing medicines with family members or consuming leftover medicines stored at home [2,3]. Many drugs being used for self-medication are over-the-counter (OTC) drugs [4]. OTC drugs are meant for self-medication and are of proven efficacy and safety. However, their improper use due to insufficient knowledge about their side effects and interactions can have adverse outcomes [5]. The problems due to self-medication are augmented when non-OTC drugs are also used for self-medication as not all drugs used for self-medication are OTC drugs [5,6]. World Health Organization (WHO) advises the rational practice of self-medication which can help to prevent and manage ailments that do not require medical consultation and provides a cheaper alternative for treating common illnesses [7,8]. However, it is also known that self-medication requires information in regard to health conditions and an understanding of disease and medication.

Being future doctors, self-medication has a notable effect on MBBS students. Due to their requirement to study the details of various drugs and diseases, including their administration, side effects, contraindications, and interactions, they are more inclined toward self-medication, facilitated by their convenient and comprehensive access to drug information. This differentiation from the general population is a significant aspect. While some MBBS students become more cautious in practicing self-medication, recognizing the potential harm of irrational and inappropriate drug use, they prefer consulting qualified doctors even for minor illnesses. However, certain students, bolstered by their textbook knowledge, may become confident and initiate self-medication. Consequently, they may experience success and be encouraged to continue this approach, even providing advice to fellow medical students. On the other hand, adverse outcomes can occur, leading to detrimental health or a diseased state. Proper sensitization and awareness about self-medication among MBBS students can help mitigate these unforeseeable situations. Therefore, the present study was conducted to sensitize and raise awareness among MBBS students, examining its influence on their knowledge, attitude, views, and self-medication practices.

## Materials and methods

This was a cross-sectional, questionnaire-based study that was completed in six months (June-November 2022). This study was conducted on MBBS students at an Autonomous Medical College in Bihar after obtaining permission from the ethics committee of the Institute (vide letter no. 1491/IEC/IGIMS). A self-designed, pre-validated questionnaire consisting of close-ended questions was used which was divided into the following parts: Part A - Demographic details of the participants (Appendix: Figure 2), Part B - Knowledge of participants on self-medication (Appendix: Figure 3), Part C - Attitude of participants on self-medication (Appendix: Figure 4, Table 1), Part D - Views of participants on self-medication (Appendix: Figure 4, Table 2) and Part E - Practice of participants on self-medication (Appendix: Figure 5).

All the students were made aware of this study and students who were willing to participate were included. Students who were not willing were excluded. The study was conducted on Phase I (first year) and Phase II (second year) students during their scheduled lecture/practical class and Phase III (third and fourth year) students were involved through online platforms (WhatsApp Group, Google Forms, and Zoom Cloud Meeting) to conduct this study. A brief description of the nature of the study, its purpose, and the procedure of completing and submitting the questionnaire was explained to the participants through their respective mediums. Written informed consent was obtained from every student before filling the questionnaire and confidentiality was ensured to them. The questionnaire was then circulated to the participating students and their responses were collected/recorded. The incompletely filled-up questionnaires were excluded and finally, 267 responses were used for data interpretation scores were given to each student on the basis of their response submitted in “knowledge aspect of self-medication.” On the basis of this score, each student was given a grade (Table 1). 

**Table 1:** Grading scale for knowledge aspect of self-medication

Grade	Score
A	8-10
B	5-7
C	3-4
D	0-2

After collecting the responses, a proper sensitization and awareness program was conducted for these participating students through interactive lectures, group dynamics activities along with role play and seminars using audio-visual aids through the respective medium. After three months of educational activities the same questionnaire (Parts B, C, and D) was distributed again among the participating students, and later on, their responses were collected. With an expected 40% prevalence of self-medication in a total of 439 students, the minimum sample size needed to attain a power of 95% and an alpha value of 0.05 was calculated to be 201. Statistical Package for Social Sciences (SPSS) version 16 was used for data analysis. The survey was descriptive and qualitative data were described as numbers and percentages. The age of participants was assessed in terms of mean and standard deviation (SD). The chi-square test was used to see the association in responses obtained, before and after sensitization. A p-value with ≤0.05 was considered statistically significant.

## Results

The questionnaires were circulated among 439 students, out of which 314 responses were obtained. However, 47 of these responses were excluded due to its incompleteness, resulting in 267 valid responses for data interpretation. Among these 267 responses, 158 (59.1%) were from male participants, while 109 (40.8%) were from female participants. The mean age of the participants was 22.23 ± 2.37 years, as shown in Table 2.

**Table 2:** Demographic variables

Characteristics	Values (n=267)	Percentage
Age (Mean ± SD)	22.23 ± 2.37	
Sex		
Male	158	59.18
Female	109	40.82
Residence		
Urban	115	43.07
Semi-urban	34	12.73
Rural	18	6.74
Health Perception		
Good	171	64.04
Excellent	53	19.85
Could be better	43	16.1
Health protective measure (eating healthy food)		
Yes	219	82.02
No	48	17.98
Health protective measure (doing regular exercise)		
Yes	110	41.2
No	157	58.8
Last use of healthcare service		
Doesn’t remember	52	19.48
30-60 days	38	14.23
2-6 months	37	13.86
6-12 months	79	29.59
More than 1 year	61	22.85
Recent history of self-medication		
Yes	234	87.6
No	33	12.3

**Figure 1:**
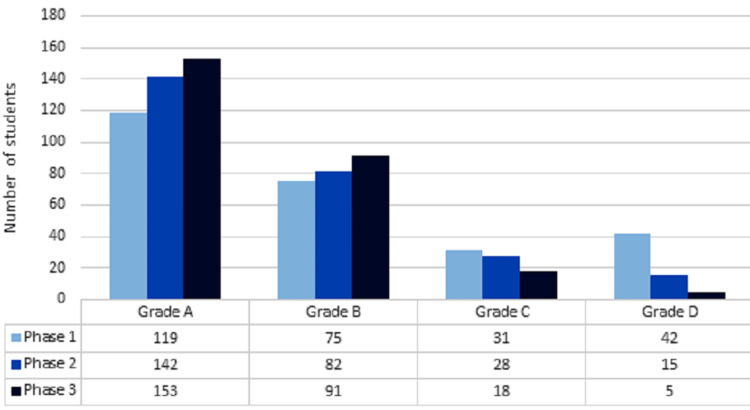
Grades of knowledge on self-medication among students from different phases Scores were given to each student on the basis of their response submitted in “knowledge aspect of self-medication.” On the basis of this score, each student was given a grade.

**Figure 2:**
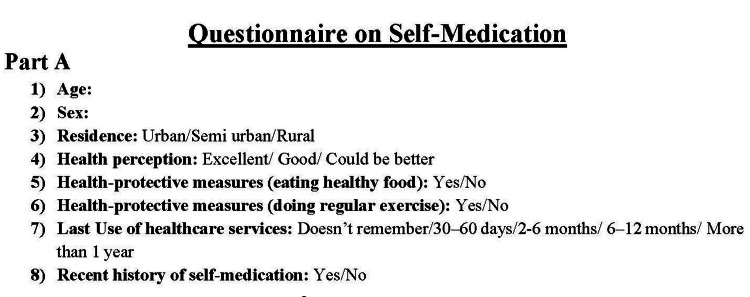
Questionnaire on Self-Medication - PART A

**Figure 3:**
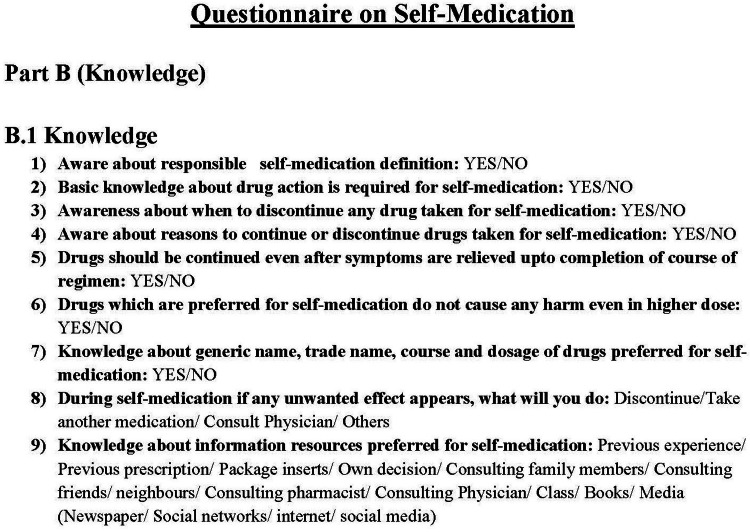
Questionnaire on Self-Medication - PART B (Knowledge)

**Figure 4:**
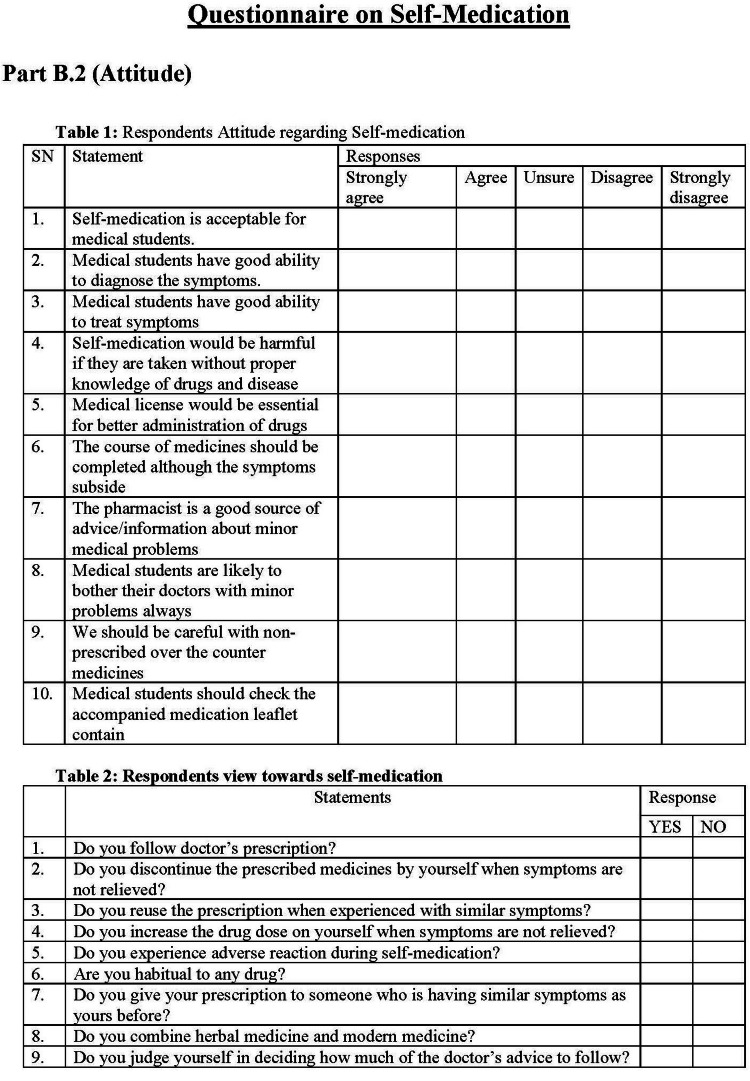
Questionnaire on Self-Medication - PART B (Attitude)

**Figure 5:**
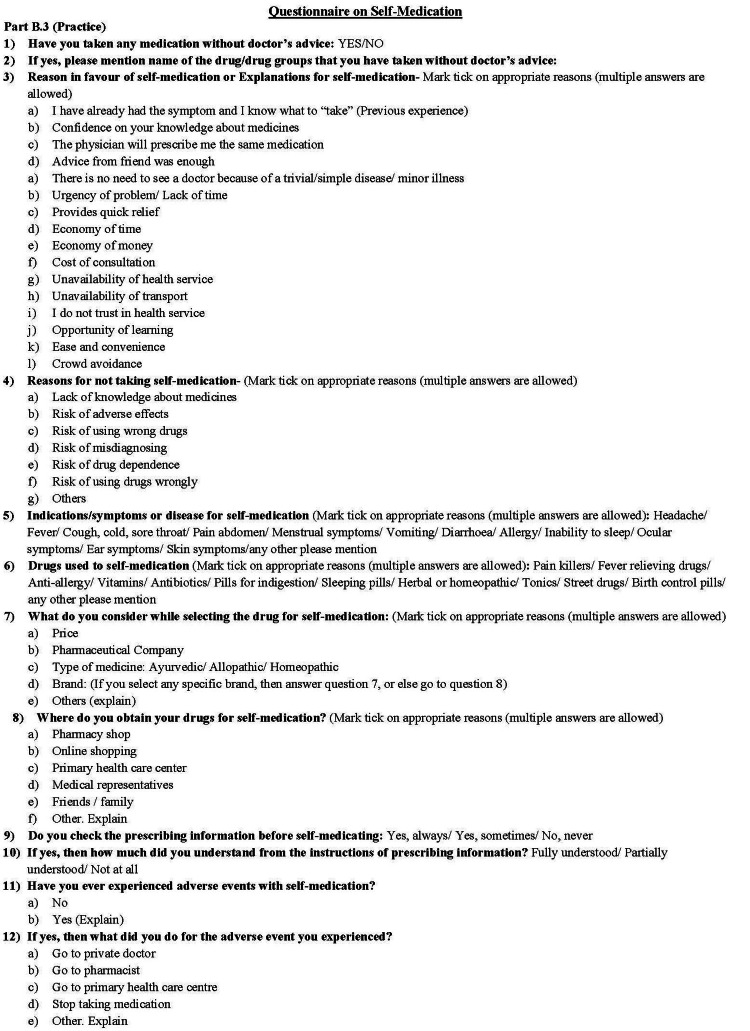
Questionnaire on Self-Medication PART B (Practice)

Scoring was done for each participating students on the basis of their response regarding knowledge about self-medication before conducting educational activity. On the basis of scoring, grades were given to different phases of students as shown in Figure 1.

On analyzing the knowledge aspect, we found that good number of students were aware about responsible self-medication definition and this awareness increased after conducting educational activities as shown in Table 3.

**Table 3:** Knowledge of participants on self-medication before and after educational activities

Knowledge Aspect		Response	Before educational activities	After educational activities	P-value (Chi-Square
re about responsible self-medication definition		Yes	211	239	0.0008
		No	56	28	
Basic knowledge about drug action is required for self-medication		Yes	241	255	0.01
		No	26	11	
Awareness about discontinuation of drug taken for self-medication		Yes	226	245	0.01
		No	41	22	
Aware about reasons to continue or discontinue drugs taken for self-medication		Yes	199	221	0.02
		No	68	46	
Drugs should be continued even after symptoms are relieved up to course of regimen		Yes	203	223	0.03
		No	64	44	
Drugs which are preferred for self-medication do not cause any harm even in higher dose		Yes	45	26	0.02
		No	222	241	
Knowledge about generic name, trade name, course and dosage of drugs preferred for self-medication		Yes	173	194	0.049
		No	94	73	
Action after unwanted effects of self-medication	Discontinue		187	46	<0.0001
	Take another medication		16	12	
	Consult Physician		64	209	
Information resources preferred for self-medication: Ticking multiple option is allowed	Class/ Books		38	41	0.09
	Previous experience		116	109	
	Consulting Physician		83	101	
	Consulting Family Members		11	03	
	Media (Newspaper/ Social networks/ internet/ social media)		19	13	

The grades obtained by students based on scores given to them in knowledge aspect was compared as shown in Table 4.

**Table 4:** Comparison of grades obtained by students based on scores in knowledge aspect

Grade	Before educational activities	After educational activities	P-value (Chi-Square)
Grade A (Score 8-10)	157	207	<0.0001
Grade B (Score 5-7)	39	33	
Grade C (Score 3-4)	48	21	
Grade B (Score 0-2)	23	6	

On analyzing the attitude aspect, we noted that good number of students agreed that self-medication is acceptable for medical students however after carrying the educational activity they disagreed to this aspect as shown in Table 5.

**Table 5:** Attitude of participants on self-medication before and after educational activities

Attitude Aspects		Choice					P-value (Chi-Square)
		Strongly Agree	Agree	Unsure	Disagree	Strongly Disagree	
Self-medication is acceptable for medical students	Before Educational Activity	11	139	53	49	15	0.04
	After Educational Activity	9	118	48	80	12	
Medical students have good ability to diagnose the symptoms	Before Educational Activity	26	94	98	30	19	0.09
	After Educational Activity	20	88	84	50	26	
Medical students have good ability to treat symptoms	Before Educational Activity	23	83	113	41	8	0.04
	After Educational Activity	15	81	102	64	16	
Self-medication would be harmful if they are taken without proper knowledge of drugs and disease	Before Educational Activity	169	86	4	4	4	0.01
	After Educational Activity	202	57	2	1	1	
Medical license would be essential for better administration of drugs	Before Educational Activity	124	109	11	19	4	0.001
	After Educational Activity	154	98	4	5	1	
The course of medicines should be complete although the symptoms subside	Before Educational Activity	120	113	19	8	8	0.04
	After Educational Activity	151	98	8	5	6	
The pharmacist is a good source of advice/information about minor medical problems	Before Educational Activity	26	113	56	38	34	<0.001
	After Educational Activity	1	11	16	98	141	
Medical students are likely to bother their doctors with minor problems always	Before Educational Activity	23	75	113	45	11	0.005
	After Educational Activity	6	71	117	41	24	
We should be careful with non-prescribed over the counter medicines	Before Educational Activity	102	109	45	8	4	0.03
	After Educational Activity	113	125	22	6	2	
Medical students should check/read the accompanied medication leaflet/package insert	Before Educational Activity	98	132	19	11	8	0.01

On looking into the view aspect, it was noted that participants had view that self-medication can lead to adverse reactions. This view was more pronounced after carrying the educational activity as shown in Table 6.

**Table 6:** View of participants on self-medication before and after educational activities

Views	Response	Before educational activities	After educational activities	P-value (Chi-square)
Follow doctor’s prescription	Yes	259	266	0.02
	No	8	1	
Discontinue the prescribed medicines by yourself when symptoms are not relieved	Yes	83	58	0.01
	No	184	209	
Reuse the prescription when experienced with similar symptoms	Yes	158	129	0.01
	No	109	138	
Increase the drug dose on yourself when symptoms are not relieved	Yes	34	16	0.007
	No	233	251	
Can experience adverse reaction during self-medication?	Yes	192	244	< 0.0001
	No	75	23	
Can become habitual to any drug with self-medication	Yes	38	27	0.14
	No	229	240	
Give your prescription to someone who is having similar symptoms as yours before	Yes	60	42	0.047
	No	207	225	
Combine herbal medicine and western medicine	Yes	90	63	0.01
	No	177	204	
Judge yourself in deciding how much of the doctor’s advice to follow	Yes	120	89	0.006
	No	147	178	

The practice aspect showed that an important reasons in “for” of self-medication were that it is easy and convenient, economy of time and money and no need to see a doctor for minor illness as shown in Table 7.

**Table 7:** Practice aspect of self-medication

Reason in for of self-medication		
Reasons	Number of students	Percentage of students
I have already had the symptom and I know what to “take” (Previous experience)	124	46.44
Confidence on your knowledge about medicines	117	43.82
The physician will prescribe me the same medication	139	52.06
Advice from friend was enough	26	9.74
There is no need to see a doctor because of a trivial/simple disease/ minor illness	165	61.80
Urgency of problem/ Lack of time	139	52.06
Provides quick relief	184	68.91
Economy of time	196	73.41
Economy of money	181	67.79
Cost of consultation	128	47.94
Unavailability of health service	117	43.82
Unavailability of transport	109	40.82
I do not trust in health service	26	9.74
Opportunity of learning	139	52.06
Ease and convenience	211	79.03
Crowd avoidance	150	56.18
Reasons in against of self-medication		
Reasons	Number of students	Percentage of students
Lack of knowledge about medicines	207	77.53
Risk of adverse effects	221	82.77
Risk of using wrong drugs	221	82.77
Risk of misdiagnosing	224	83.89
Risk of drug dependence	169	63.30
Risk of using drugs wrongly	226	84.64
Others	150	56.18
Condition/Symptom for self-medication		
Condition/Symptom	Number of students	Percentage of students
Headache	222	83.15
Fever	226	84.64
Cough, cold, sore throat	203	76.03
Pain abdomen	165	61.80
Menstrual symptoms	124	46.44
Vomiting	196	73.41
Diarrhoea	184	68.91
Allergy	169	63.30
Inability to sleep	60	22.47
Ocular symptoms	49	18.35
Ear symptoms	56	20.97
Skin symptoms	86	32.21
Prophylaxis for certain disease or infection	49	18.35
Drug/Group of Drugs used for self-medication		
Drugs/Group of Drugs	Number of students	Percentage of students
Pain killers	213	79.77
Fever relieving drugs	224	83.89
Anti-allergy	188	70.41
Vitamins	214	80.15
Antibiotics	154	57.68
Pills for indigestion	173	64.79
Sleeping pills	60	22.47
Herbal or homeopathic	128	47.94
Tonics	124	46.44
Street drugs	45	16.85
Birth control pills	86	32.21
Antiviral drugs	49	18.35
Antifungal drugs	102	38.20
Anti-amoebic drugs	71	26.59
Criteria for selecting drugs for self-medications		
Criteria	Number of students	Percentage of students
Price	109	40.82
Pharmaceutical Company	173	64.79
Type of medicine: Ayurvedic/ Allopathic/ Homeopathic	213	79.77
Brand	135	50.56
Other	68	25.47
Place of obtaining drugs for self-medications		
Place	Number of students	Percentage of students
Pharmacy shop	232	86.89
Online shopping	71	26.59
Primary health care centre	90	33.71
Medical representatives	98	36.70
Friends / family	135	50.56
Other	23	8.61
Checking prescribing information (from leaflet inside medicine package)		
Always checked	173	64.79
Checked it sometime	86	32.21
Never checked	8	3.00
Understanding prescribing information		
Fully understood	128	47.94
Partially understood	131	49.06
Never understood	8	3.00
Action after adverse effect of self-medications		
Action	Number of students	Percentage of students
Consulting physician	181	67.79
Going to pharmacist	45	16.85
Consulting primary health care centre	135	50.56
Stop taking medication	188	70.41
Others	41	15.36

## Discussion

Several studies have previously been conducted on self-medication, but no studies have been conducted in India to analyze the role of sensitization and awareness programs on the knowledge, attitude, views, and practice of self-medication among MBBS students. Hence, this study was conducted to examine the impact of educational activities on the perception of self-medication. Participation in this study was voluntary and included MBBS students from all three phases (first year to fourth year). All the participants were adolescents, and a male preponderance was observed. Studies on self-medication have reported various prevalence figures, ranging from 43.2% to 91% [9-12]. This variation may be attributed to the diverse demography, socioeconomic status, and availability of non-prescription drugs over the counter. In our study, 87.6% of the participants had a history of self-medication. On proportional analysis, it was observed that out of 158 male participants, 104 (65.8%) had a history of self-medication, while out of 109 female participants, 91 (83.4%) had a history of self-medication. Other studies have also reported a higher number of females engaging in self-medication compared to males [13,14].

Scoring was done for each participating student on the basis of their response regarding knowledge about self-medication before conducting the educational activity. On the basis of scoring, grades were given to different phases of students which were then compared. In comparison, we found that the phase 3 students (third and fourth year) comparatively had better knowledge regarding different aspects of self-medication. This may be due to their more academic knowledge and clinical exposure in contrast to students of phases 1 and 2. There is a correlation between the year of study and the level of knowledge about self-medication, which can be interpreted that “knowledge increases with the level of education” as also reported by Gyawali et al. and Shankar et al. [15,16].

On analyzing the knowledge aspect, we found that a good number of students were aware of responsible self-medication definition and this awareness increased after conducting educational activities which was statistically found to be highly significant. In addition, we also found that the majority of participants had the perception that “basic knowledge about drug action is required for self-medication.” However, after sensitization through educational activities, this perception changed which was statistically found to be significant. Further, it was seen that participants had the knowledge that “if they experience any unwanted effects of self-medication” then they should “discontinue the current medication for its cure.” This knowledge changed to “consulting physician” after conducting educational activity which was statistically found to be highly significant.

On analyzing the attitude aspect, we noted that a good number of students agreed that self-medication is acceptable for medical students; however, after carrying out the educational activity they disagreed with this aspect which was statistically significant. In addition, the majority of participants agreed that a “pharmacist is a good source of advice/information about minor medical problems” which has also been reported by Mehta et al. and Wajantri et al. [17,18]. However, post-sensitization they strongly disagreed with this, and the difference was found to be highly significant.

Upon examining the viewpoint, it was observed that participants held the belief that self-medication can result in adverse reactions. This perspective became more prominent after conducting the educational activity, which was statistically highly significant.

Further, the practice aspect was analyzed under different parameters. The important reasons “for” self-medication were that it is easy and convenient, the economy of time and money, and no need to see a doctor for minor illnesses. These perceptions are akin to those outlined by WHO that self-medication provides a cheaper and more convenient alternative for treating common minor illnesses [7]. In addition, similar reasons had been reported by other studies also [19-22]. Similarly, important reasons “against” self-medication were the risk of misdiagnosing and adverse effects which has been previously reported by James et al. and Hughes et al. [14,19]. This may be due to the knowledge about adverse drug reactions among the participants. On further analysis, it was seen that fever followed by headache were the most common causes of self-medication, and drugs relieving fever (antipyretic-analgesic) were the most common group of drugs used. A similar pattern of self-medication had been reported by other studies also [23-27].

The place of obtaining drugs showed that the majority of participants opted pharmacy shop and this can be co-related to their previous response which showed that the majority of participants had the attitude that “pharmacist is a good source of advice/information about minor medical problems.” This reason was followed by obtaining drugs from family/friends as procuring becomes easy if any family member/ friend is a doctor which has been also acclaimed by Gyawali et al. [15]. Upon experiencing adverse effects followed by self-medication majority of the participants had practice of stopping the medication followed by consulting the physician. This practice can be validated as it is consistent with their response given before sensitization in the knowledge aspect.

There are certain limitations of the present study. Although the respondents were requested to complete the questionnaire independently, the mutual influence between the students could not be entirely ruled out. We could not analyze the practice aspect after educational activities. This could be done in the future with a longer duration of study. The results of the study would have been more generalized per se if it could involve students from other medical colleges in Bihar. The strength of our study was its method, as we tried to spread awareness regarding different aspects of self-medication, and we also analyzed its impact statistically. To the best of our knowledge, no studies have been done in India in this context which has been a demand from previous studies [15,27]. In addition, a good sample size was also the strength of our study as 71.5% of MBBS students from our institute turned up voluntarily to participation. The response rate and its completeness were apt as it was found to be 85%.

## Conclusions

Healthcare providers, who possess knowledge about diseases and medications, are more susceptible to self-medication, which can even influence the non-medical population. MBBS students, as future doctors, should be well-informed about the pros and cons of self-medication. This study demonstrated that participating students' perceptions regarding various aspects of self-medication significantly improved after our educational activities. We anticipate that these young aspiring doctors, once aware, will disseminate this knowledge to benefit society. Additionally, we hope that this study will have an impact on students from different medical colleges and even healthcare providers, promoting responsible self-medication practices when necessary.

## References

[1] (2023). Guidelines for the regulatory assessment of medicinal products for use in self-medication. https://apps.who.int/iris/handle/10665/66154.

[2] Elmahi OK, Musa RA, Shareef AA (2022). Perception and practice of self-medication with antibiotics among medical students in Sudanese universities: a cross-sectional study. PLoS One.

[3] (2023). The role of the pharmacist in self-care and self-medication: report of the 4th WHO consultative group on the role of the pharmacist. https://apps.who.int/iris/handle/10665/65860.

[4] Hernandez-Juyol M, Job-Quesada JR (2002). Dentistry and self-medication: a current challenge. Med Oral.

[5] Goh LY, Vitry AI, Semple SJ, Esterman A, Luszcz MA (2009). Self-medication with over-the-counter drugs and complementary medications in South Australia's elderly population. BMC Complement Altern Med.

[6] Sherazi BA, Mahmood KT, Amin F, Zaka M, Riaz M, Ahmed J (2012). Prevalence and measure of self-medication: a review. J Pharm Sci Res.

[7] (2023). Report of the WHO Expert Committee on National Drug Policies, Geneva, 19-23 June 1995: contribution to updating the WHO guidelines for developing national drug policies. https://apps.who.int/iris/handle/10665/63068.

[8] Shah R, Ramanuj V, Bala DV (2015). Evaluation of self-medication among urban population of Paldi area, Ahmedabad. Natl J Community Med.

[9] Gutema GB, Gadisa DA, Kidanemariam ZA (2011). Self-medication practices among health sciences students: the case of Mekelle University. J Applied Pharmaceutical Sci.

[10] Girish HO, Divya HM, Prabhakaran S, PPV PPV, Koppad R, Acharya A (2013). A cross-sectional study on self-medication pattern among medical students at Kannur, North Kerala. J Evol Med Dent Sci.

[11] Patel P, Prajapati AK, Ganguly B, Gajjar B (2013). Study on impact of pharmacology teaching on knowledge, attitude and practice on self-medication among medical students. Int J Med Sci Public Health.

[12] Kumar N, Kanchan T, Unnikrishnan B (2013). Perceptions and practices of self-medication among medical students in coastal South India. PLoS One.

[13] Klemenc-Ketis Z, Hladnik Z, Kersnik J (2011). A cross sectional study of sex differences in self-medication practices among university students in Slovenia. Coll Antropol.

[14] James H, Handu SS, Al Khaja KA, Otoom S, Sequeira RP (2006). Evaluation of the knowledge, attitude and practice of self-medication among first-year medical students. Med Princ Pract.

[15] Gyawali S, Shankar PR, Poudel PP, Saha A (2015). Knowledge, attitude and practice of self-medication among basic science undergraduate medical students in a medical school in western Nepal. J Clin Diagn Res.

[16] Shankar PR, Dubey AK, Dwivedi NR, Nandy A, Barton B (2016). Knowledge, perception and practice of self-medication among premedical and basic science undergraduate medical students. Asian J Med Sci.

[17] Mehta RK, Sharma S (2015). Knowledge, attitude and practice of self-medication among medical students. IOSR J Nursing Health Sci.

[18] Wajantri P, Angadi MM, Masali KA, Masali KA, KJ S, Bhat S, Jose AP (2013). Study on knowledge, attitude and practice about self medication among college students. Int J Health Sci Res.

[19] Hughes CM, McElnay JC, Fleming GF (2001). Benefits and risks of self medication. Drug Saf.

[20] Shankar PR, Partha P, Shenoy N (2002). Self-medication and non-doctor prescription practices in Pokhara valley, Western Nepal: a questionnaire-based study. BMC Fam Pract.

[21] Banerjee I, Bhadury T (2012). Self-medication practice among undergraduate medical students in a tertiary care medical college, West Bengal. J Postgrad Med.

[22] Badiger S, Kundapur R, Jain A (2012). Self-medication patterns among medical students in South India. Australas Med J.

[23] Goel D, Gupta S (2013). Self-medication patterns among nursing students in North India. J Dental Med Sci.

[24] Hanumaiah V, Harini M (2018). Study of knowledge, attitude and practice of self-medication among health care workers at MC Gann Teaching District hospital of Shivamogga, India. Int J Basic Clin Pharmacol.

[25] Sundararajan A, Thangappan AK (2018). Knowledge, attitude and practice of self-medication among undergraduate medical students in a teaching institution. Int J Basic Clin Pharmacol.

[26] Kayalvizhi S, Senapathi R (2010). Evaluation of the perception, attitude and practice of self-medication among business students in 3 select cities, South India. IJEIMS.

[27] Abay SM, Amelo W (2010). Assessment of self-medication practices among medical, pharmacy, and health science students in Gondar 
University, Ethiopia. J Young Pharm.

